# Supplementary material for: NicheProt: Cell-type-resolved proteomics of tissue compartments
Source: Sci Adv. 2026 Jul 15;12(29):eaee6237. doi: 10.1126/sciadv.aee6237 (PMC13371888; doi:10.1126/sciadv.aee6237)
Supplement: Supplementary file 1 — Figs. S1 to S3 Tables S1 and S2 [file sciadv.aee6237_sm.pdf]

Science Advances
AAAS

Supplementary Materials for

NicheProt: Cell-type-resolved proteomics of tissue compartments

Yi-Chien Wu *et al.*

Corresponding author: Steve Seung-Young Lee, ssylee@uic.edu

Sci. Adv. **12**, eaee6237 (2026)
DOI: 10.1126/sciadv.aee6237

This PDF file includes:

Figs. S1 to S3
Tables S1 and S2

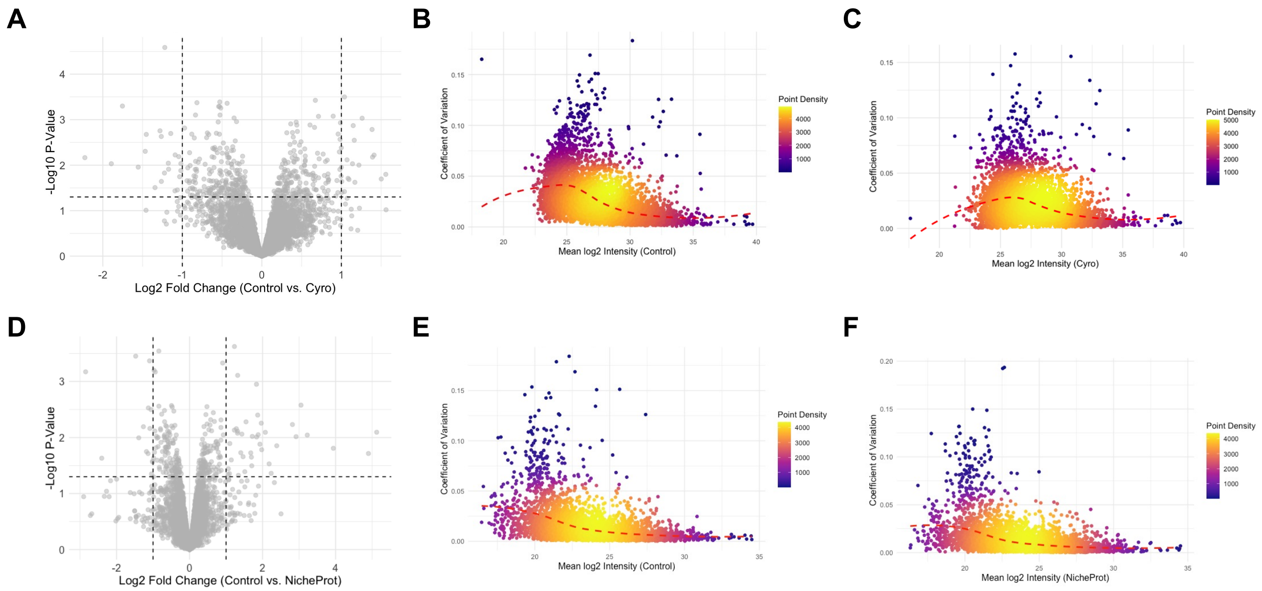

Fig. S1. Quantitative assessment of the proteome in control and NicheProt samples. (**A**) Volcano plot comparing control and cryopreserved samples. (**B** and **C**) Coefficients of variation (CVs) within control (B) and cryopreservation (C) groups were below 0.2, demonstrating high reproducibility between biological replicates. (**D**) Volcano plot comparing control and NicheProt samples showed no significant protein expression changes. (**E** and **F**) CVs plot within the control (E) and NicheProt (F) conditions were below 0.2.

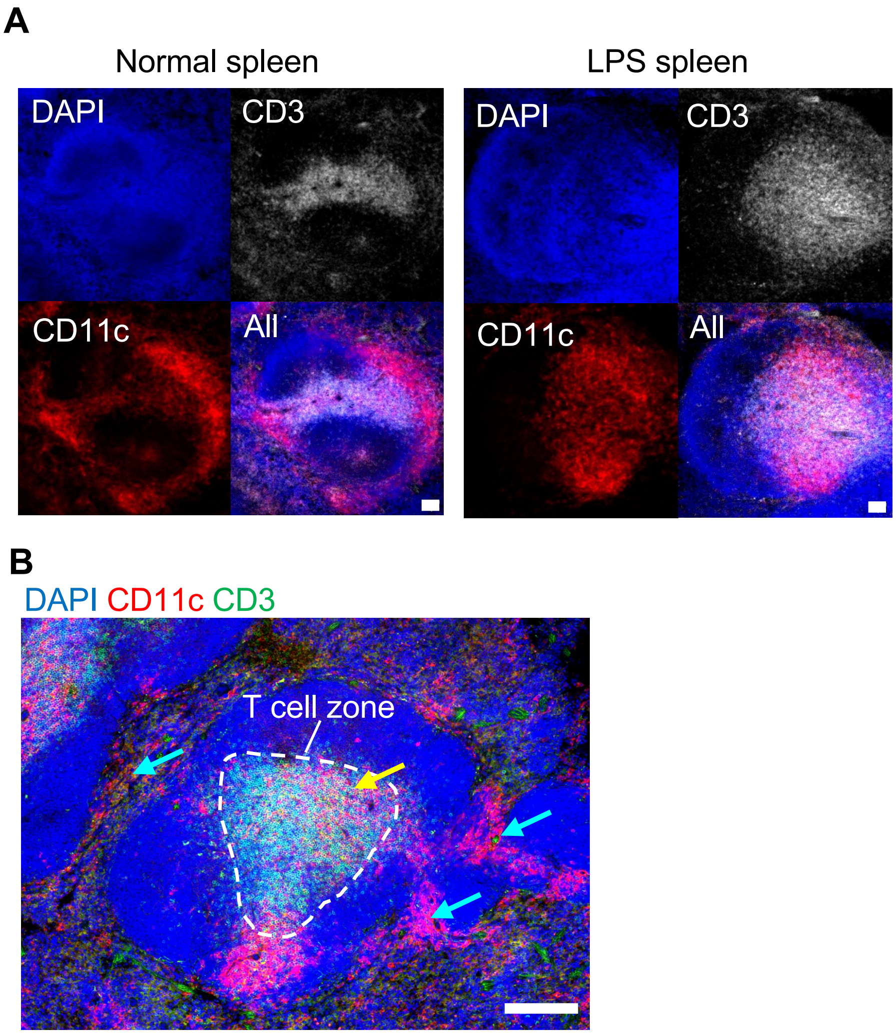

Fig. S2. Dendritic cell migration during LPS-induced inflammation. (**A**) Splenic dendritic cells (DCs) primarily reside outside the T cell zone (TCZ) in a normal spleen. DCs migrate toward the TCZ upon LPS-induced inflammation. DCs were labeled with Alexa Fluor 647-anti-CD11c antibody, while T cells were stained with DyLight 550-anti-CD3 antibody. Scale bars: 50 µm. (**B**) During inflammation, there are two DC populations located inside (yellow arrow) and outside (cyan arrows) the TCZ. Scale bar: 100 µm.

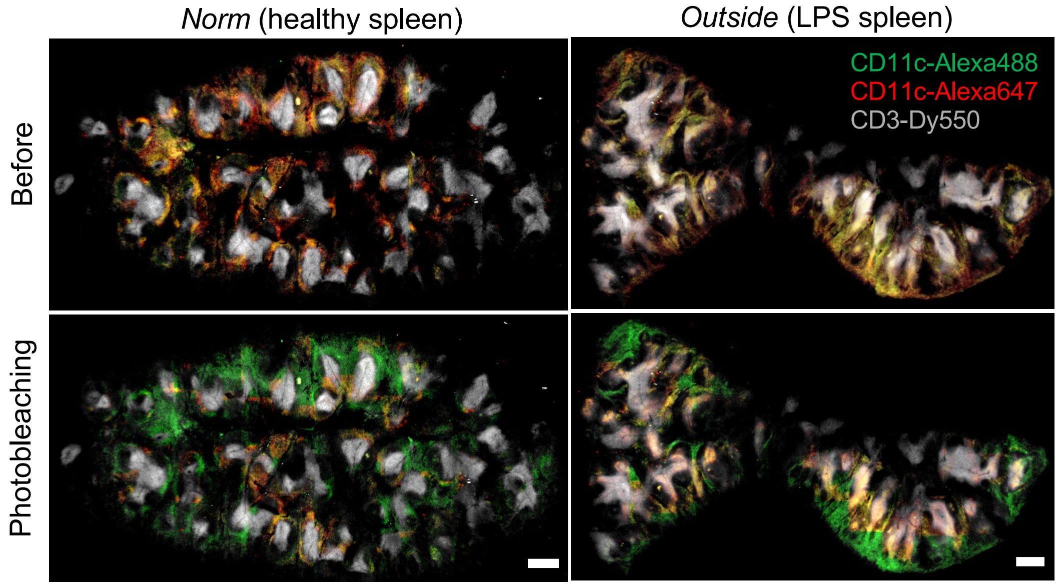

Fig. S3. Photobleaching-mediated barcoding of DCs outside the TCZs in the (left) healthy and (right) LPS-treated mouse spleen macrosections. Tissue macrosections were labeled with Alexa Fluor 488-anti-CD11c (green), Alexa Fluor 647-anti-CD11c (red), and Dylight-CD3 (gray). Photobleaching of Alexa Fluor 647 on CD11c^{+} DCs outside the CD3^{+} TCZs converted yellow signals to green. Scale bar: 500 μm.

Table S1. The volumes, estimated cell numbers, and protein amounts of 3D photobleached regions in mouse spleen macrosections using 20× and 40× objectives with varying zoom factors.

Objective	Zoom factor	x (μm)	y (μm)	z (μm)	Volume (μm^3)	Estimated cell number (10 areas)	Estimated protein (μg) (10 areas)
20x air	1.5x	610	500	400	1.22E+08	4.12E+05	12.31
	2x	450	400	400	7.20E+07	2.43E+05	7.26
	3x	300	260	400	3.12E+07	1.05E+05	3.14
	4x	220	225	400	1.98E+07	6.69E+04	2.00
40x air	3x	230	170	400	1.56E+07	5.29E+04	1.58
	4x	125	110	400	5.50E+06	1.86E+04	0.56

Table S2. Antibody information

Antibody	Fluorescence	Clone	Source	Antibody concentration	Dye to antibody ratio
**Anti-CD3**	DyLight 550	500A2	Biolegend	0.5 mg/ml	30:1
**Anti-CD45**	DyLight 550	30-F11	Biolegend	0.5 mg/ml	30:1
**Anti-CD163**	DyLight 550	S150491	Biolegend	0.5 mg/ml	20:1 or 30:1
**Anti-Fscn1**	Goat-Anti-rabbit-Alexa Fluor 488	Polyclonal	ProteinTech	0.3 mg/ml	N/A
**Anti-Sh3bp1**	Goat-Anti-rabbit-Alexa Fluor 488	Polyclonal	ProteinTech	0.6 mg/ml	N/A
**Anti-GzmB**	FITC	NGZB	Invitrogen	0.5 mg/ml	Commercially conjugated antibody
**Anti-Pecam1**	Alexa Fluor 488	MEC13.3	Biolegend	0.5 mg/ml	Commercially conjugated antibody
**Anti-CD11c**	Alexa Flour 488	N418	Biolegend	0.5 mg/ml	Commercially conjugated antibody
	Alexa Flour 647				
